# Zipper Pattern: An Investigation into Psychotic Criminal Detection Using EEG Signals

**DOI:** 10.3390/diagnostics15020154

**Published:** 2025-01-11

**Authors:** Gulay Tasci, Prabal Datta Barua, Dahiru Tanko, Tugce Keles, Suat Tas, Ilknur Sercek, Suheda Kaya, Kubra Yildirim, Yunus Talu, Burak Tasci, Filiz Ozsoy, Nida Gonen, Irem Tasci, Sengul Dogan, Turker Tuncer

**Affiliations:** 1 Department of Psychiatry, Elazig Fethi Sekin City Hospital, Elazig 23280, Turkey; akcagulay01@gmail.com (G.T.); suheda_sener@hotmail.com (S.K.); 2 School of Business (Information System), University of Southern Queensland, Toowoomba, QLD 4350, Australia; prabal.barua@usq.edu.au; 3 Department of Digital Forensics Engineering, Technology Faculty, Firat University, Elazig 23119, Turkey; 212144203@firat.edu.tr (D.T.); tkeles@firat.edu.tr (T.K.); 231144106@firat.edu.tr (S.T.); isercek@firat.edu.tr (I.S.); kubra.yildirim@firat.edu.tr (K.Y.); 210509039@firat.edu.tr (Y.T.); 210509033@firat.edu.tr (N.G.); turkertuncer@firat.edu.tr (T.T.); 4 Vocational School of Technical Sciences, Firat University, Elazig 23119, Turkey; btasci@firat.edu.tr; 5 Department of Psychiatry, School of Medicine, Tokat Gaziosmanpasa University, Tokat 60100, Turkey; filiz.ozsoy@gop.edu.tr; 6 Department of Neurology, School of Medicine, Firat University, Elazig 23119, Turkey; itasci@firat.edu.tr

**Keywords:** zipper pattern, psychotic criminal detection, EEG signal processing, digital forensics, neuro forensics, explainable feature engineering, tkNN, directed Lobish

## Abstract

**Background:** Electroencephalography (EEG) signal-based machine learning models are among the most cost-effective methods for information retrieval. In this context, we aimed to investigate the cortical activities of psychotic criminal subjects by deploying an explainable feature engineering (XFE) model using an EEG psychotic criminal dataset. **Methods:** In this study, a new EEG psychotic criminal dataset was curated, containing EEG signals from psychotic criminal and control groups. To extract meaningful findings from this dataset, we presented a new channel-based feature extraction function named Zipper Pattern (ZPat). The proposed ZPat extracts features by analyzing the relationships between channels. In the feature selection phase of the proposed XFE model, an iterative neighborhood component analysis (INCA) feature selector was used to choose the most distinctive features. In the classification phase, we employed an ensemble and iterative distance-based classifier to achieve high classification performance. Therefore, a t-algorithm-based k-nearest neighbors (tkNN) classifier was used to obtain classification results. The Directed Lobish (DLob) symbolic language was used to derive interpretable results from the identities of the selected feature vectors in the final phase of the proposed ZPat-based XFE model. **Results:** To obtain the classification results from the ZPat-based XFE model, leave-one-record-out (LORO) and 10-fold cross-validation (CV) methods were used. The proposed ZPat-based model achieved over 95% classification accuracy on the curated EEG psychotic criminal dataset. Moreover, a cortical connectome diagram related to psychotic criminal detection was created using a DLob-based explainable artificial intelligence (XAI) method. **Conclusions:** In this regard, the proposed ZPat-based XFE model achieved both high classification performance and interpretability. Thus, the model contributes to feature engineering, psychiatry, neuroscience, and forensic sciences. Moreover, the presented ZPat-based XFE model is one of the pioneering XAI models for investigating psychotic criminal/criminal individuals.

## 1. Introduction

Violence is defined as any destructive behavior with the explicit purpose of causing harm [1,2]. The World Health Organization, on the other hand, defines “violence” as any behavior that results in injury, death, or mental problems [3]. The relationship between violence and psychiatric disorders has been investigated for many years. In some studies, it has been reported that the tendency towards violence both towards oneself and towards the outside (other people, objects, or animals) increases in psychiatric disorders [4,5]. In some of these studies, it has been reported that destructive acts of violence and self-mutilation attempts against oneself increase [1,4]. Again, in some studies, higher rates of violent tendency and involvement in crime were found in individuals with psychiatric disorders compared with the general population [6,7]. In these studies, it was not reported that the tendency to violence increased in all psychiatric disorders [8], but the rates of violent tendency and involvement in crime increased in some psychiatric disorders such as bipolar disorder, schizophrenia (SZH), schizoaffective disorder, and alcohol/substance use disorders [9]. Studies on the etiology of psychiatric disorders and violent tendency are limited in the literature [10,11,12,13]. In one of these studies, it was shown that factors such as the age, gender, marital status, and socioeconomic status of the patient may increase the tendency to violence [13]. In another study, it was reported that patients’ treatment non-compliance or alcohol/substance use may increase the tendency to violence [10]. In recent years, non-invasive methods that also show functional and structural changes in the central nervous system have been utilized to investigate the etiology of psychiatric disorders [14,15,16]. Electroencephalography (EEG) is one of these methods. EEG is a non-invasive method that is widely used in the evaluation of electrical activities in the brain. By interpreting EEG signals, brain activity can be automatically detected [15,16]. The interpretation of EEG signals is used to identify psychiatric disorders such as depressive disorder, bipolar disorder, and SZH, in addition to neurological diseases [16,17,18,19,20]. However, the relationship between EEG signals and the tendency to commit crime or violence in psychiatric diseases has not been examined in the literature [21]. In the light of all this information, in our study, we aim to automatically detect and classify the psychiatric diseases we have previously identified using EEG signals, and then to obtain brain-based findings of psychiatric patients involved in crime with a new artificial intelligence-based symbolic language.

### 1.1. Related Works

In this section, some related works are presented. Park et al. [22] aimed to classify major psychiatric disorders with EEG data. The retrospective data of 945 participants were analyzed with resting-state EEG measurements, intelligence scores, and clinical diagnoses. Support vector machines, random forest, and elastic network methods were compared; the elastic network model showed the highest performance in accuracy. Accuracy was reported as 93.83% for SZH, 91.21% for trauma and stress disorders, and 74.52% for obsessive–compulsive disorder. A limitation of this study is that the results were not validated in large groups of participants. The findings suggest that machine learning (ML) with EEG can provide objective diagnostic tools. Machetanz et al. [23] proposed a ML-based model to predict adverse treatment processes in forensic patients with SZH. The retrospective dataset of 356 patients included 209 parameters, from which the 10 most influential variables were identified. Variable reduction was performed in data balancing and model selection. The gradient boosting algorithm showed the best performance with 67% balanced accuracy and 72% AUC. The model was able to predict negative treatment processes with factors such as antisocial behavior, physical violence, and the need for isolation. The limitations of this study include the retrospective nature of the data and the limited sample size. Bhattacharya et al. [24] examined the opportunities and challenges of artificial intelligence (AI) applications for neuro-prediction and violence risk assessment in forensic psychiatry. Using secondary data analysis, this study addressed methods to predict potential violent and recidivist criminal behavior by combining neuroimaging with AI algorithms. ML models can be used to categorize individuals into low and high risk groups. Among the limitations of this study, ethical and legal concerns were highlighted; for example, risks such as algorithmic biases and privacy violations were noted. Srinivasan et al. [25] presented a novel method for improving SZH diagnosis using EEG data. Data were collected from a dataset of 81 participants (36 SZH and 22 healthy controls). The proposed method optimized EEG signal preprocessing and classification by integrating a deep learning architecture based on a Convolutional Neural Network (CNN) and Long Short-Term Memory (LSTM) with a mutation-assisted Archimedean Optimization (MAO) algorithm. Performance metrics such as 98.2% accuracy, 97.2% F1 score, 98.9% sensitivity, and 98.5% precision were achieved. Chen et al. [26] investigated the discriminability of SZH, psychotic major depression (PMD), and non-psychotic major depression (NPMD) using resting-state EEG dynamic functional connectivity analysis. Their study included 579 participants (197 healthy controls, 152 NPMD, 45 PMD, and 185 SCZ patients), and static and dynamic functional connectivity features and classification algorithms were applied on EEG data. The dynamic functional connectivity model successfully distinguished the four groups with an accuracy of 73.1%, outperforming the static connectivity model, which achieved an accuracy of 49.3%. However, this study is limited by the small sample size for the PMD group and the heterogeneity resulting from single-center data. Zhou et al. [27] present a method to investigate methamphetamine abusers using EEG signals. Their study analyzed EEG signals from 18 female methamphetamine abusers and 22 healthy controls, focusing on P300 components for feature extraction. Their model achieved a classification accuracy of 83.85%. Pettorruso et al. [28] aimed to develop a machine learning-based system using EEG and clinical data for the treatment of refractory depression. This system, called SelecTool, aimed to determine personalized treatment options using clinical criteria together with data from EEG and biomarkers. In the first phase, 100 treatments of refractory depression patients were randomly assigned to esketamine nasal spray or accelerated repetitive transcranial magnetic stimulation treatments, and in the second phase, the model was validated with 20 participants. Preliminary results showed the potential of the model to improve treatment response rates. The limitations of this study are the small sample size and single-center data. Masuda et al. [29] aimed to classify human fear levels into four categories with a deep learning model based on EEG and environmental physiological signals. Multi-channel EEG and environmental physiological signals from the DEAP dataset were used and a Multi-Input CNN LSTM model was proposed. This model showed the highest performance with 98.79% accuracy and 99.01% F1 score. The limitation of the study is the imbalanced dataset and the effect of individual differences on classification performance. Baumgartl et al. [30] developed an ML model based on resting-state EEG data to detect antisocial personality disorder. EEG signals collected from 84 participants (42 healthy controls and 42 antisocial personality disorder patients) were divided into 99 frequency sub-bands and analyzed using the Random Forest algorithm. The model performed with 77.5% balanced accuracy, 80% sensitivity, and 75% specificity. The limitations of this study include the small sample size and the lack of validation with other mental disorders with similar symptoms. Dongen et al. [31] discussed the potential contributions and limitations of neuroscientific approaches in forensic psychiatry and psychology. The neurobiological bases of antisocial and criminal behavior, brain regions, neurotransmitters, hormones, and various factors such as genetic predisposition were examined. In particular, it was emphasized that functional disorders in brain regions such as the orbitofrontal cortex and amygdala and serotonin–testosterone imbalances are associated with antisocial behavior. The study also states that neuroscience can be used in risk assessment and interventions, but there are ethical and methodological problems in transferring group-level findings to individual judgments.

### 1.2. Literature Gaps

The identified gaps in the literature based on the surveyed literature are as follows:

- Most research has focused on achieving high classification performance on EEG signal datasets [32]. To this end, deep learning (DL) architectures have been widely employed [33,34]. However, while DL models often achieve high accuracy, they come with drawbacks such as exponential time complexity and reliance on expensive hardware or cloud services, making them less suitable for lightweight intelligent applications [35]. This underscores the need for efficient and accurate feature engineering models [36].
- In addition to classification performance, interpretability/explainability is a critical aspect that has received limited attention in the literature [36]. Despite advances in artificial intelligence, explainable artificial intelligence (XAI) methods remain underexplored, particularly in applications related to EEG signal processing.
- There is a lack of AI-based psychotic criminal detection models in the literature.

### 1.3. Motivation and Study Outline

Individuals who commit crimes during psychotic episodes are defined as those diagnosed with a severe mental illness characterized by psychotic symptoms such as delusions or hallucinations, those who have been incarcerated for criminal behavior, are undergoing treatment in a high-security forensic psychiatric facility, or possess a documented history of criminal offenses [37,38]. The primary objective of this research is to investigate the brain structures of these individuals and address gaps in the existing literature. To fill the given gaps in the literature, a new EEG psychotic criminal dataset was curated because a publicly available EEG psychotic criminal dataset could not be found, and a ZPat-related explainable feature engineering (XFE) model was presented.

To address the first gap in the literature, a highly accurate feature engineering model was needed. Therefore, we were motivated to present a new generation channel-based feature extraction function, termed Zipper Pattern (ZPat). The presented ZPat extracts relationships between qualified channels, and its primary purpose is to extract the most meaningful features and capture hidden patterns. By deploying the proposed ZPat, a new XFE model was developed with a cognitive structure.

In the feature selection phase, an iterative feature selector was needed to capture the most informative features. Thus, iterative neighborhood component analysis (INCA) [39,40] was utilized. The INCA feature selector is a self-organized, distance-based feature selector. Based on the INCA, a highly accurate classifier was required. Therefore, an ensemble distance-based classifier, specifically a t-algorithm-based k-nearest neighbors (tkNN) [41], has been used to yield high classification performance. Since the tkNN classifier is a distance-based, self-organized classifier, the presented ZPat-based XFE model attained high and reliable classification performance using this cognitive approach.

To address the second gap, the DLob-based XAI method was integrated into the presented ZPat-based model. This integration converted the feature engineering model into an XFE model. Moreover, a DLob string and a DLob-related cortical connectome diagram were generated for psychotic criminal/criminal detection, and brain-based findings were extracted.

In the literature, there are limited psychiatry-related XAI works. To fill this gap, we curated a new EEG psychotic criminal detection dataset, and a ZPat-based XFE model was presented.

### 1.4. Innovations and Contributions

The innovations and contributions of the recommended work are listed below.

Innovations:

- A new EEG psychotic criminal dataset was curated. In this regard, we have created a new testbed for psychotic criminal detection.
- A new generation channel-based feature extraction function has been presented. The introduced feature extractor is termed ZPat.
- A new ZPat-based XFE model has been developed.
- To the best of our knowledge, the recommended ZPat-based XFE model is the first XFE model for psychotic criminal/criminal detection.

Contributions:

- The presented ZPat-based XFE model has a cognitive structure designed to yield high classification accuracy. In this work, the model achieved over 95% classification accuracy with LORO and 10-fold CVs. Thus, the ZPat-based XFE model is a highly accurate feature engineering model with linear time complexity. In this respect, this research contributes to machine learning.
- By integrating the DLob symbolic language, explainable results have been obtained from the identities of the selected features. A cortical connectome diagram has been generated using DLob. In this context, the introduced ZPat-based XFE model contributes to medical sciences, providing insights into the psychotic criminal/criminal brain by extracting a cortical map. Moreover, this model also contributes to forensic science.

## 2. Materials and Methods

### 2.1. Materials

To create a new testbed for psychotic criminal/criminal detection, an EEG dataset was curated. These EEG signals were collected from participants in two categories: (i) psychotic criminal and (ii) control. The psychotic criminal EEG signals were collected from individuals hospitalized in a high-security forensic psychiatric hospital. The control group was composed of non-criminal volunteers. To collect the signals, some mafia-related video content was shown to the participants. This dataset was collected from 130 participants. The EEG signals were collected over a longer duration for the control group. The dataset includes 37 (29 male + 8 female) healthy non-criminal controls and 27 psychotic criminals (25 male + 2 female). The age range of the control participants is 19 to 47, while the age range of the psychotic criminal participants is 22 to 59.

For the dataset collection process, an Emotiv Flex EEG cap was used. This brain cap has 32 channels, which are as follows: (i) Cz, (ii) Fz, (iii) Fp1, (iv) F7, (v) F3, (vi) FC1, (vii) C3, (viii) FC5, (ix) FT9, (x) T7, (xi) CP5, (xii) CP1, (xiii) P3, (xiv) P7, (xv) PO9, (xvi) O1, (xvii) Pz, (xviii) Oz, (xix) O2, (xx) PO10, (xxi) P8, (xxii) P4, (xxiii) CP2, (xxiv) CP6, (xxv) T8, (xxvi) FT10, (xxvii) FC6, (xxviii) C4, (xxix) FC2, (xxx) F4, (xxxi) F8, and (xxxii) Fp2. The sampling frequency of this brain cap is 256 Hz.

In this dataset, the duration of each EEG segment is 15 s. Consequently, the length of each EEG segment is 3840 × 32, where 32 refers to the number of channels. The distribution of the collected EEG psychotic criminal dataset is depicted in Table 1.

Table 1 demonstrates that the curated EEG psychotic criminal detection dataset is an unbalanced dataset.

### 2.2. Zipper Pattern

**Figure 1:**
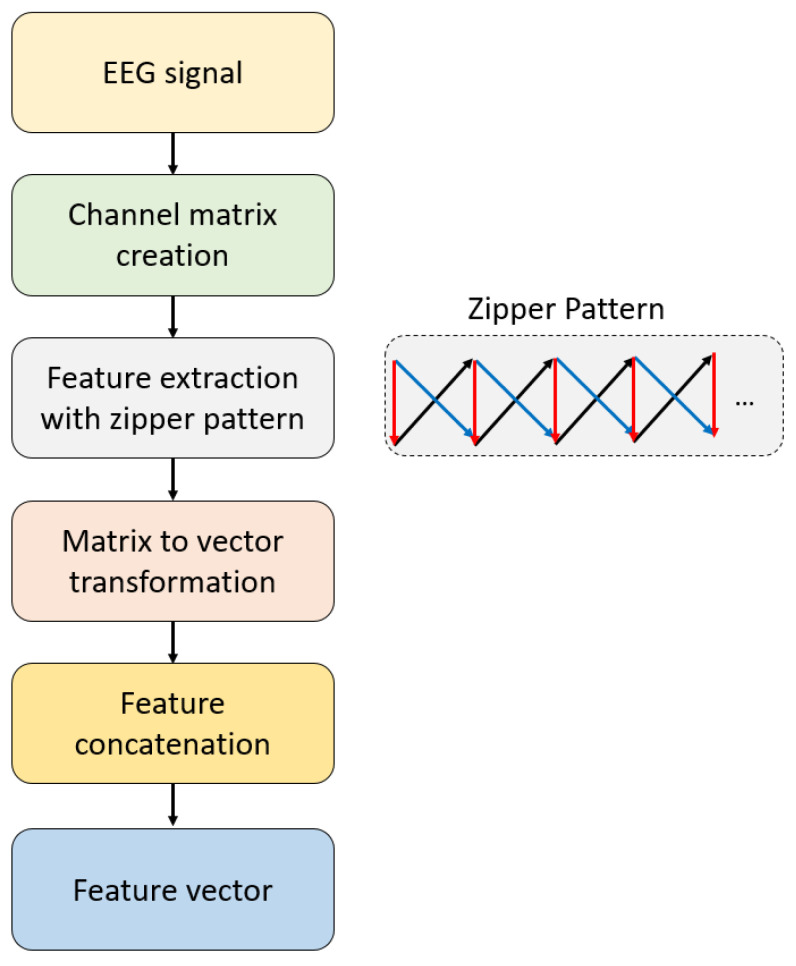
The schematic overview of the recommended ZPat feature extractor.

The essential objective of the recommended ZPat is to generate meaningful features from EEG signals and to obtain explainable results using the features generated by ZPat. Therefore, we have presented a new channel-based feature extractor, and the graphical overview of the recommended ZPat feature extractor is showcased in Figure 1.

To better clarify the recommended ZPat, the steps of this feature extractor have been given below.

Step 1: Generate overlapped channel matrices.

(1) $${cm}^{i}=EEG\left(i:i+1,j\right),i\in \left\{1,\;2,\ldots ,L-1\right\},\;j\in \left\{1,\;2,\ldots ,32\right\}$$

where cm is the channel matrix with a size of 2 × 32, EEG is the curated psychotic criminal EEG signal, and L is the length of the EEG signal.

Step 2: Sort the channel matrix and obtain the qualified identities.

(2) $$id_{t}^{i}=argsort\left(-cm^{i}\left(:,t\right)\right),\;t\in \left\{1,2\right\}$$

where id is the qualified indices in descending

Step 3: Compute three transition tables using the Zipper Pattern. Here, the main objective is to capture the differences of the channel ordering.

(3) $${tt}_{r}=\left[\begin{array}{ccc} 0 & \cdots & 0\\ \vdots & \ddots & \vdots \\ 0 & \cdots & 0 \end{array}\right],r\in \left\{1,2,3\right\}$$

(4) $${tt}_{1}\left(id_{1}^{i}\left(a\right),id_{2}^{i}\left(a+1\right)\right)={tt}_{1}\left(id_{1}^{i}\left(a\right),id_{2}^{i}\left(a+1\right)\right)+1,\;a\in \left\{1,2,\ldots ,31\right\}$$

(5) $${tt}_{2}\left(id_{2}^{i}\left(a\right),id_{1}^{i}\left(a+1\right)\right)={tt}_{2}\left(id_{2}^{i}\left(a\right),id_{1}^{i}\left(a+1\right)\right)+1$$

(6) $${tt}_{3}\left(id_{1}^{i}\left(b\right),id_{2}^{i}\left(b\right)\right)={tt}_{3}\left(id_{1}^{i}\left(b\right),id_{2}^{i}\left(b\right)\right)+1,b\in \left\{1,2,\ldots ,32\right\}$$

Herein, tt is the transition table and three transition tables have been created in this step. Moreover, the size of each transition table is 32 × 32.

Step 4: Repeat steps 1–3 until the required number of matrices is reached, and complete the creation of these three transition tables for the EEG signal used.

Step 5: Concatenate the three generated transition tables in vector form.

(7) $$feat\left(g+32^{2}\left(r-1\right)\right)=tt_{r}\left(q,w\right),\left(q,w\right)\in \left\{1,2,\ldots ,32\right\},g\in \left\{1,2,\ldots ,32^{2}\right\}$$

where feat is the generated feature vector with a length of 3072 (=32 × 32 × 3).

The given five steps above have defined the introduced ZPat feature extractor.

The 2 × 32 matrix size represents the dimensions of the channel matrices created during the feature extraction process. Specifically, the ZPat feature extractor considers overlapping EEG channel pairs, generating matrices where the rows correspond to two consecutive channels and the columns represent the 32 EEG channels recorded from the cap, with 32 being the total number of channels. To minimize complexity, the feature extraction process is performed using the smallest possible matrix size. The method employs three transition matrices to capture different types of channel transitions. The first matrix extracts features by monitoring changes in the order of adjacent channels over time. The second matrix identifies features related to reverse-order transitions between channels. The third matrix encodes static relationships between paired channels. These transition matrices enable the creation of a richer and more comprehensive feature set. Specifically, temporal features capture changes in channel orderings, reflecting the dynamic structure of neural activity. Statistical features are naturally encoded within the transition matrices, including the frequency and probability of specific channel transitions. Additionally, ordering and transition-based coding captures nonlinear interactions between channels, which are often characteristic of complex brain activity.

In this context, the presented ZPat feature extractor transforms raw EEG signals into a compact and interpretable feature set. Each feature vector in this set includes channel information, and channel differences are easily computed. In this regard, the extracted feature set is well-suited for both machine learning and XAI applications.

### 2.3. The Recommended XFE Model

**Figure 2:**
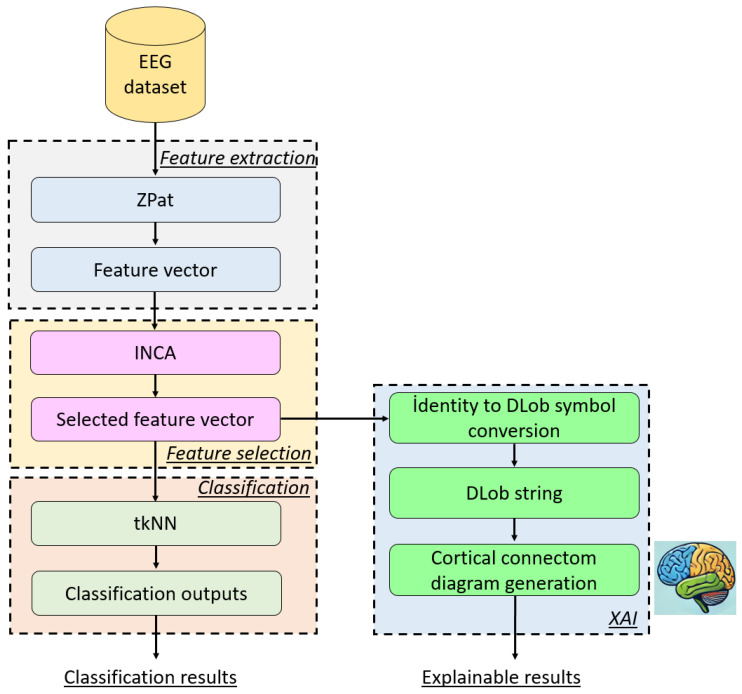
The schematic overview of the presented ZPat-based XFE model.

In this research, we have presented a new generation XFE model, with the major goal of obtaining both high classification accuracy and explainable results. To achieve this, we introduced a new cognitive approach for achieving high classification performance. Additionally, DLob [42] has been integrated into this model to provide explainable results related to psychotic criminal detection. The introduced ZPat-based XFE model generates valuable insights into the detection of the psychotic criminal brain. To better explain the presented ZPat-based XFE model, a graphical depiction of this model is shown in Figure 2.

Figure 2 clearly showcases that the presented ZPat-based XFE model has four phases. In the first phase, 3072 features are extracted from each EEG signal using the introduced ZPat feature extractor. In the feature selection phase, the most informative features are selected using the INCA feature selector, and these selected features are utilized to obtain both explainable and classification results. To obtain the classification results, the tkNN classifier is used, with the selected features serving as input. The last phase is the XAI generation phase, where the identities of the selected features are used to create a DLob string to generate explainable results. To better define the recommended ZPat-based XFE model, the definitions of this model are provided phase by phase below.

Phase 1 (Feature extraction with ZPat): Extract 3072 features from each EEG sample using the recommended ZPat feature extractor.

(8) $$X\left(n,1:3072\right)=ZPat\left(EEG_{w}\right),n\in \{1,2,\ldots ,Dm\}$$

Herein, X is the created feature matrix, ZPat(.) is the introduced ZPat feature extractor, and Dm is the number of EEG samples.

Phase 2 (INCA-based feature selection): Select the most meaningful features by deploying INCA feature selectors.

(9) $$[ind,CX]=INCA\left(X,y\right)$$

Here, ind are the indices of the selected features (CX), INCA(.) is the INCA feature selection function, and y is the actual label.

The INCA feature selector was proposed by Tuncer et al. [39] in 2020. The primary objective of this model is to select the most informative features from the available features. Herein, the process involves NCA [43], a feature selection loop, evaluation of the selected features’ classification ability, and greedy algorithm-based selection of the best feature vector. The definitions of these steps are as follows.

In the first step, the qualified indices (ix) of the features have been computed by the NCA feature selector (NCA(.)).

(10) ix=NCA(X,y)

In the second step, a loop has been defined to apply the iterative feature selection process.

(11) $$sf^{s-start+1}\left(n,h\right)=X\left(n,ix\left(h\right)\right),\;s\in \left\{start,start+1,stop\right\},\;h\in \left\{1,2,\ldots ,s\right\}$$

where sf is the iteratively selected feature vector, start is the start value of the loop, and stop is the stop value of the loop.

In the third step, the classification accuracies of all selected feature vectors have been computed by deploying a classifier. For this research, a kNN classifier [44] has been utilized to compute the classification accuracies of the selected feature vectors.

(12) $$ca\left(u\right)=kNN\left(sf^{u},y\right),\;s\in \left\{1,,start+1,stop-start+1\right\}$$

Herein, ca is the classification accuracy and kNN(.) is the kNN classifier.

In the fourth (last) step, the best selected feature vector has been chosen as the final selected feature vector deploying the greedy algorithm [45].

(13) sx=argmax(ca)

(14) $$CX=sf^{sx}$$

Above, the greedy algorithm has been defined and the maximum accurate selected feature vector has been chosen as the final selected feature vector. In this aspect, the recommended INCA feature selector is a self-organized feature selector.

The feature selection phase is a crucial step for both classification and the generation of explainable results. By using the selected features as input to the tkNN classifier, the classification results are generated. By linking the selected features to symbolic representations of the DLob, the model provides interpretable results. For example, it may indicate which brain regions are most active or how the hemispheric transitions contribute to psychotic criminal symptoms.

Phase 3 (Classification): Obtain the classification results by deploying the tkNN [41,46] classifier.

(15) cot=tkNN(CX,y)

Herein, cot is the classification output and tkNN(.) is the tkNN classifier. Two validation techniques have been used for obtaining classification results and these are (i) LORO CV and (ii) 10-fold CV. The used tkNN classifier has three steps as follows:

- Parameter-based outcome generation: In this step, an iterative parameter changing has been applied to the kNN classifier and more than one parameter-based outcome has been generated. For this study, the changed parameters are (i) distances, (ii) weights, and (iii) k values. By changing these parameters, 60 parameter-based outcomes have been created.
- Majority voting: in order to created voted results, iterative majority voting (IMV) [47] has been used and 58 voted outcomes has been created from the generated 60 outcomes.
- Greedy algorithm: In this work, the generated 118 (=60 + 58) outcomes have been selected using the maximum classification accuracy. In this step, the maximum accurate outcome has been selected as the final outcome.

In these regards, kNN is straightforward and suitable for small-scale or less complex problems, while tkNN introduces iterative parameter tuning, voting, and optimization to obtain more powerful and reliable classifiers for complex datasets requiring higher accuracy and robustness. tkNN is the improved, ensemble, and iterative version of kNN.

Phase 4 (DLob-based XAI): Extract interpretable results by using the identities of the selected features and DLob [42] symbolic language.

(16) $${sy}_{g}=\left(\left\lfloor \frac{ind\left(p\right)}{32^{2-g}}\right\rfloor \;\left(mod\;32\right)\right)+1,\;g\in \{1,2\}$$

(17) $$DlS\left(c+g-1\right)=LUT\left({sy}_{g}(j)\right),c\in \{1,3,\ldots ,sc-1\}\;$$

where sy is the index value of the look-up-table (LUT) generated from the selected features, DlS is the generated DLob sequence, and sc is the symbol count. The used LUT has been defined below.

LUT: {Cz, Fz, FL, FL, FL, FLCL, CL, FLCL, FLTL, TL, CLPL, CLPL, PL, PL, PLOL, OL, Pz, Oz, OR, PROR, PR, PR, CRPR, CRPR, TR, FRTR, FRCR, CR, FRCR, FR, FR, FR}. This LUT has been defined using the channels of the 32-channel brain cap utilized in the study. As seen from the LUT, some channels are associated with two symbols. Therefore, each selected feature contains information corresponding to 2, 3, or 4 DLob symbols.

The phases described above define the presented ZPat-based XFE model for EEG signal classification.

## 3. Experimental Results

The results of the recommended ZPat-based XFE model are presented in this section. Firstly, to implement the recommended model, a simply configured laptop was utilized. The ZPat-based model was developed using the MATLAB (version 2024a) programming environment. Parallel programming and graphical/tensor processing units were not used to implement the model, as it was a lightweight model. For implementation, only the central processing unit (CPU) mode was used.

The recommended ZPat-based XFE model is a parametric model, and the parameters used are tabulated in Table 2.

By using these parameters, the presented ZPat-based XFE model has been implemented.

The first evaluation parameter is the time complexity. To evaluate the time complexity analysis, big O notation has been used and the time complexity analysis of the presented model is given below.

The ZPat feature extractor has been used to extract features and the time complexity of this extractor is O(L), where L defines the length of the EEG signal.

The INCA feature selector has used NCA, an iteration, and a kNN classifier. In this aspect, the time complexity of the INCA is equal to O(N + RK). Herein, N is the time complexity coefficient of the NCA feature selector, R is the range of the iteration, and K is the time complexity coefficient of the kNN classifier.

tkNN has been utilized as the main classifier. In this classifier, parameter-based and voted outcomes have been generated. At this point, the time complexity of the tkNN classifier is equal to O(PK + V), where P is the number of the parameters and V is the number of the voted outcomes.

In the last phase, DLob-based interpretable results are generated. To obtain the explainable results, a DLob string was created and it was generated using the selected features. Therefore, the time burden of this phase is computed as O(S). Herein, S is the number of the selected features.

In this aspect, the total time complexity of the presented ZPat-based XFE model is equal to O(L + N + RK + PK + V + S). This time burden demonstrates that the introduced ZPat-based model has linear time complexity.

**Figure 3:**
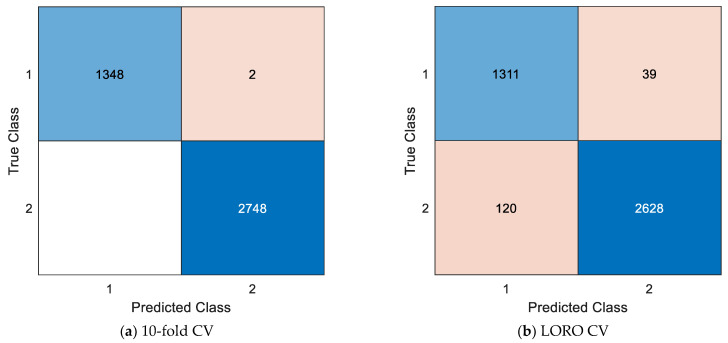
The computed confusion matrices with (**a**) 10-fold CV and (**b**) LORO CV. Herein, 1: Psychotic criminal, 2: Control.

In the first evaluation metric, the presented ZPat-based XFE model has linear time complexity. The second evaluation metric is the classification performance since we have presented an EEG signal classification model. In order to evaluate the classification performance of the recommended model, the commonly used classification performance evaluation metrics have been used and these are as follows: (i) classification accuracy, (ii) sensitivity, (iii) specificity, (iv) precision, (v) F1 score, and (vi) geometric mean. Moreover, 10-fold CV and LORO CV techniques have been utilized. To compute these classification metrics, two confusion matrices were computed and the computed confusion matrices are demonstrated in Figure 3.

Per Figure 3, the computed classification metrics have been listed in Table 3.

These results clearly demonstrate that the recommended model achieved over 90% accuracy for both validation techniques, with 10-fold CV attaining higher classification performance than LORO CV. In 10-fold CV, the same record’s EEG segment can be included in both training and testing, which may lead to higher classification results compared with LORO CV. However, due to the structure of the dataset, where the ’Psychotic Criminal’ class contains 93 records from 27 participants, there is potential confusion regarding the independence of EEG signals. To address this, we prioritized the LORO CV method, ensuring that EEG signals from the same participant were left out during validation to minimize overlap and evaluate the model’s robustness.

The third performance evaluation metric is the explainable results. To obtain interpretable results, a DLob sentence was created, and the information entropy and transition table of the generated DLob sentence were computed. The obtained DLob sentence is provided below.

The DLob sentence generated for psychotic criminal detection:

“FRFRFLFRCLCRPRFLCLFLFLCROzTRTROzOzOzCRFzPLFLFLPRPROzPRFLCLFLCLPzPRPLFzFLCLCLPLCLCLPLFLORCLOLFzFzCRPRCLPRPLCLPLPzPzOzPzPRFRFRFLPRORPLPLOzCLFLTLPLPLPROzCRCLPLFLCLPLFLCLCRPRCLPLCLCLFLPLOLTRCzFRCRFLFLFLFRCRPRORPLFLCzCzFLCLFLCLFRCRPRPzOzCLPLPzOzCLPRPLTLTLPLPRCRPRCLTLTLFLTLFLTLFRTRPLFLTLFRTRPRFRFRCLPLCLPLPRPzCRCRFLTLPLTLFLORCLTRTRFRTRPRFLFLPRPRFRFRFRCRFRCRPzPRFLCLTRFLTLPLPLOLPLOLPRPRCzCzFRCRFRCROzCRFRCRTLTRFLCLFLTLFLTLFLFRPzPzOzOzFLTLFRTRFLCLPLCRPRCLPLFzFLFRCRPRORTRTRCLPLFLCLFLCLCLPLOzPRTLFRFzPLCLPLCLFLCLFLCLFLPRORTLFLPRPLFLPLFLPLOLFRPRFLFLFzPLFLPLCLFLCRPRFLFLPLCzCzPRFzFRPRFLFLTLPLPRCLFRTRPzPzPRFLCLPLCLPLFRCRFLCLORFRFRPLPLFRTRFRTRFLPLFRCRFRFLPLPLFzFRTLPLPLFRTRFLTLCLFRTRFLCLFRFzFLCRPRFRFLTLFLFRFRPRFLFRCRTLFLTLFLPRPRFLCzFLCRPRTLTLFLPRFLTLFLFLFLFzFzCzFzFLCLFLCLFRFzPRFRFLCRPRPLFzFRFRORFLFzTRFRFzFLCLFRCRPRCLFRTRFLTLCLCRPRPLPLCLPLFLOLORFLCzCRPRCLCLPLCLPLCLPLCLPLCRPRCRPRFRCRPRFLCLFLCLCzTLTRFz”

**Figure 4:**
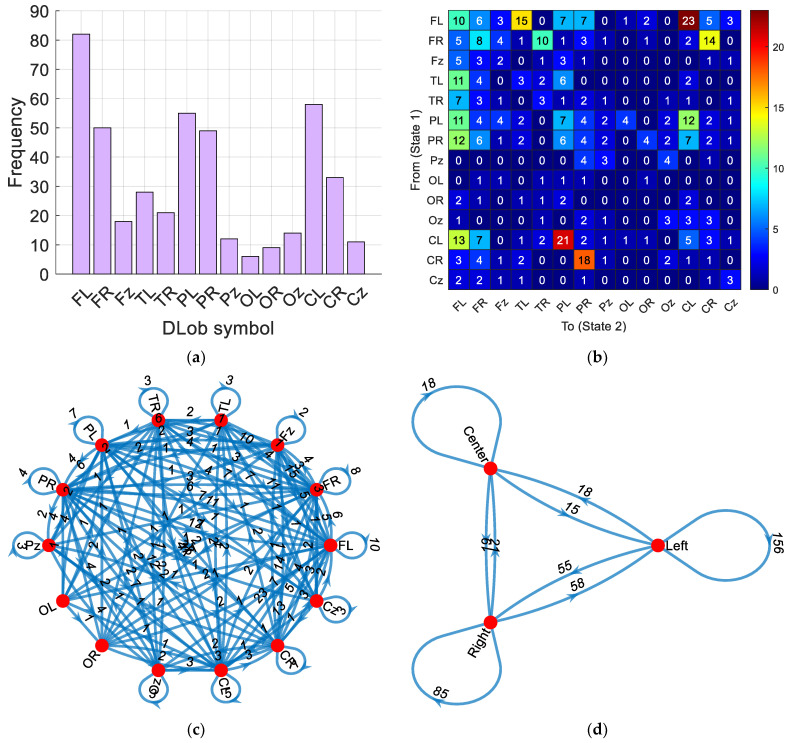
The explainable results. (**a**) Histogram of the symbols. (**b**) Transition table of the symbols. (**c**) Cortical connectome diagram. (**d**) Hemispheric cortical diagram.

By using the given DLob sentence above, we generated the histogram of the symbols, and from this histogram, the information entropy of the sentence was computed. The information entropy of this sentence was calculated as 3.4575 (close to the maximum entropy of 3.8074 = log_2_14). This information entropy reflects the complex cortical activation involved in psychotic criminal detection. The interpretable results derived from the obtained DLob sentence are depicted in Figure 4.

According to Figure 4, the obtained findings are as follows. Figure 4a shows that the most frequently used symbol is the FL symbol. Moreover, the frontal, parietal, and central lobes are more active than other lobes.

Figure 4b,c highlight that the most frequent transition is between FL and CL (23 times).

Figure 4d demonstrates that the most frequently used hemisphere is the left hemisphere for psychotic criminal detection.

## 4. Discussion

The presented ZPat-based model was evaluated using three performance evaluation metrics. The first performance evaluation metric is time burden analysis, and the given time complexity computation demonstrated that the recommended model has linear time complexity. Moreover, this model was implemented using a simply configured laptop in CPU mode. In this regard, the recommended ZPat-based model can be integrated into lightweight embedded devices.

The second performance evaluation metric is classification performance, and two validation techniques, namely 10-fold CV and LORO CV, were utilized. By deploying these validation techniques, the robustness and reliability of the presented model were demonstrated. This model yielded classification accuracies of 99.95% and 96.12% with 10-fold CV and LORO CV, respectively. The introduced ZPat-based model achieved high classification accuracy due to its self-organized nature, as both the INCA feature selector and tkNN classifier are self-organized ML methods. INCA selects the best option (selected feature vector) from 401 selected feature vectors, and tkNN chooses the best outcome from the 118 generated outcomes.

**Figure 5:**
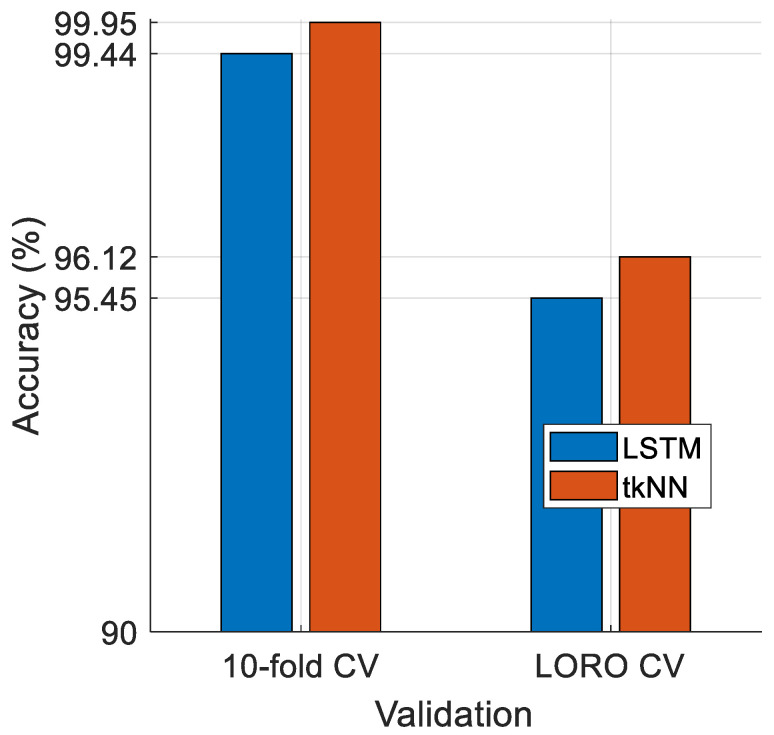
Comparison of the LSTM and tkNN classifier.

To demonstrate the superiority of the presented classification model (ZPat + INCA + tkNN), we compared the tkNN classifier with the LSTM classifier, and the results for this dataset are shown in Figure 5.

Figure 5 showcases that the tkNN classifier outperforms the LSTM classifier (a deep classifier), as the tkNN classifier achieved classification accuracies of 99.95% and 96.12% for 10-fold CV and LORO CV, respectively, while the LSTM deep classifier achieved accuracies of 99.44% and 95.45% for 10-fold CV and LORO CV, respectively. In this regard, the shallow tkNN classifier attained higher classification performance than the deep classifier for this model.

Here (see Figure 5), the LSTM classifier is a deep classifier and achieved high classification performance, while the tkNN classifier is an iterative, ensemble, and self-organized classifier. The tkNN classifier generates multiple outcomes and can automatically select the best outcome. In this regard, the tkNN classifier attained higher classification performance than LSTM, clearly demonstrating that a shallow classifier can outperform a deep classifier by utilizing the t-algorithm.

The third performance evaluation metric is the DLob-based interpretable results generation. A DLob string has been generated for psychotic criminal detection. The explanation of the generated DLob string has been analyzed below.

The sequence begins with repeated activations of the Fz and Cz symbols, indicating sustained attention and hemisphere transitions. Subsequent activations of the CL and PL symbols indicate readiness for responses to sensory information and lobe transitions. The frequency of FL activations suggests the prevalence of logical reasoning processes. The pattern of alternating activations between these regions reflects the dynamic interaction between attention, sensory processing, interhemispheric transitions, and logical reasoning.

In the context of psychology, the resulting DLob sequence can be interpreted in terms of abnormalities observed in these regions. For example, dysfunction in the frontal lobe (FL) may be associated with impaired executive functions and decision-making [48]. Changes in the parietal lobe (PL) suggest that sensory integration and spatial awareness may be affected [49]. Abnormalities in the temporal lobe (TL) may be linked to auditory hallucinations and memory impairments [50]. Central regions (Cz and CL) are crucial for representing transitions, and their dysfunctions may reflect abnormalities associated with psychotic criminal disorders [51]. Therefore, the dominance and interaction of these regions in the sequence may indicate underlying neural mechanisms related to psychotic criminal symptoms.

Moreover, the frequent transitions in the left hemisphere suggest that tasks such as language, logic, mathematics, analytical thinking, verbal memory, sequencing, and planning may be affected.

To demonstrate the success of the presented ZPat-driven model, we compared it to state-of-the-art (SOTA) models, and the comparative results are shown in Table 4.

There are various methods in the literature for classifying psychiatric disorder cases using EEG signals. Sadi Md. Redwan et al. [55] achieved 95.51% accuracy in classifying first episode psychosis patients using PSD analysis and ML methods, but the generalizability of their results was limited due to the limited sample size and lack of anatomical data. Similarly, Ko et al. [52] achieved 93.20% accuracy rates using EEG time series transformations but had difficulties in generalization capacity due to limited data sources and the small sample size. Working on a more homogeneous population, Nsugbe et al. [53] achieved 98.30% accuracy with CNN-based methods; however, factors such as the limited age range and small sample size negatively affected the generalizability of the findings. Ravan et al. [56], working on a larger dataset, classified various psychiatric disorders with 94.17% accuracy. However, limitations such as the use of the DSM III R diagnostic system and the inability to fully examine the effects of age and smoking were among the limitations of this study. Benli et al. [54] achieved an accuracy rate of 96.23% using a ciSSA-based feature extraction method and this method provided a significant success in first episode psychosis classification. However, the high computational cost and limited population diversity reduced the generalization potential of the study. Our ZPat-based XFE model provides superior results compared with these studies in the literature. Our model achieves 99.95% accuracy with 10-fold CV and 96.12% accuracy with LORO CV, surpassing the classification achievements of other studies. In particular, the proposed ZPat feature extraction method and the tkNN classifier achieved high performance by extracting meaningful features from EEG signals. Another prominent aspect of our model is the cortical and hemispheric connectivity diagrams obtained with the DLob symbolic language. This approach not only improved the classification accuracy, but also ensured the explainability of the classification process. In this respect, our study provides not only a classification tool but also an important method for understanding brain activity. Although high accuracy rates are often reported in the literature, studies have not focused enough on the explainability of the classification process. Although Nsugbe et al. [53] obtained high accuracy rates in their study, they did not analyze the explainability of the results or the interpretation of the brain activities underlying these results. Similarly, although the ciSSA-based study of Benli et al. [54] provided high accuracy, no emphasis was placed on the explainability of the classification process. In our study, the DLob-based explainable artificial intelligence (XAI) approach detected activation patterns in the frontal, parietal, and central lobes and showed that these activations overlap with aspects of psychosis associated with criminal behavior. Furthermore, the entropy value of the DLobe string was calculated as 3.4575, demonstrating the ability of our model to represent complex neural activation patterns. In conclusion, our ZPat-based XFE model differs from other works in the literature in terms of both classification performance and explainability. This work not only offers an innovative approach to ML and signal processing methods using EEG signals, but also provides a broad application potential in forensics, neuroscience, and psychiatry. In these aspects, the ZPat-based XFE model is a strong candidate for both academic research and practical applications.

Per the comparative results, the recommended ZPat-driven model achieved satisfactory classification results. Moreover, the recommended model provided interpretable results.

The most important points (findings and advantages) of this research are as follows:

- A new channel-based feature extractor, ZPat, has been presented in this research.
- By integrating INCA, tkNN, and DLob into the presented ZPat, a new XFE model has been developed.
- The recommended ZPat-based XFE model is a lightweight model as it has linear time complexity.
- In this research, two validation techniques were used in the classification phase: 10-fold CV and LORO CV.
- The introduced ZPat-driven XFE model achieved over 95% classification accuracy, attaining 99.95% with 10-fold CV and 96.64% with LORO CV. This high performance is attributed to the self-organized nature of the model, which utilizes the INCA selector and tkNN classifier.
- We compared the performance of the tkNN classifier with LSTM (a deep classifier), and tkNN attained higher classification accuracy than LSTM.
- A DLob string was generated in this research. By deploying this string, cortical and hemispheric connectome diagrams were created to provide interpretable results.
- The information entropy of the DLob string (3.4575 out of a maximum of 3.8074) reflected the complex neural activation patterns involved in psychotic criminal detection.
- According to the DLob string, the frontal, central, and parietal lobes were the most commonly activated lobes. Central lobe activations implied hemisphere transitions, coordination of muscle movements, and planning of complex movements. Frontal lobe activation was associated with impaired executive functions and decision making. Parietal lobe activity indicated altered sensory integration and spatial awareness.
- The left hemisphere was the most activated hemisphere for psychotic criminal detection. Dominance of the left hemisphere reflected challenges in language, logic, mathematics, verbal memory, and planning tasks.

Limitations:

- The gathered dataset was collected from a single medical center and includes 64 participants with 4098 EEG segments. The EEG brain cap used has 32 channels. In this regard, a more diverse and larger dataset could be collected.
- The introduced ZPat-driven model can be implemented on other EEG datasets to demonstrate its general classification ability.

Future works:

- Collaboration with more medical centers is being planned to collect larger and more diverse EEG datasets.
- The presented ZPat-driven XFE model will be tested on other EEG datasets to demonstrate the general classification ability of the model.
- New generation EEG abnormality detection assistants can be developed using the recommended ZPat-based XFE model.
- The presented model can be integrated into EEG devices to automatically create cortical and hemispheric connectome diagrams, simplifying the translation of EEG signals.
- New neuro digital forensic systems will be developed to extract behavioral analyses based on brain mapping by utilizing the recommended ZPat-driven XFE model.

## 5. Conclusions

This work showcased the efficacy of the ZPat-based XFE model for psychotic criminal detection using EEG signals. The recommended ZPat feature extractor enabled the model to generate 3072 informative features from each EEG segment, capturing meaningful relationships between channels. By utilizing INCA for feature selection and a tkNN classifier, the model provided a lightweight and self-organized approach for EEG signal classification. The presented model yielded a classification accuracy of 99.95% with 10-fold CV and 96.12% with LORO CV. The obtained high classification performances have showcased reliable performance of this model.

This EEG signal approach combined with the DLob symbolic language to create cortical and hemispheric connectome diagrams offered explainable results/insights into brain activity. The model revealed activation patterns primarily in the frontal, central, and parietal lobes, with the left hemisphere being most active. These findings align with psychotic symptoms related to impaired executive functions, altered sensory integration, and logical reasoning processes. The information entropy of the DLob string was calculated as 3.4575 (close to the maximum entropy of 3.8074), and this reflects the complex neural activation patterns underlying psychotic criminal behavior.

The proposed ZPat-driven XFE model contributes to multiple fields, including machine learning, neuroscience, and forensic sciences, by addressing the need for interpretable and accurate EEG-based classification systems. This research is directly related to forensics and serves as a pioneering effort in neuro digital forensics. Its ability to extract explainable results through DLob enhances its utility in understanding psychotic criminal behavior while offering potential for integration into lightweight embedded EEG systems.

## Figures and Tables

**Table 1:** The distribution of the collected dataset.

Class	(1) Psychotic Criminal	(2) Control	Total/Overall
Number of EEG segments	1350	2748	4098
Number of records	93	37	130
Number of participants	27 (=25 M + 2 F)	37 (=29 M + 8 F)	64 (=54 M + 10 F)
Age range	from 19 to 47	from 22 to 59	from 19 to 59

M: male, F: female.

**Table 2:** The parameters of the presented ZPat-based XFE model.

Phase	Method	Parameters
Feature extraction	ZPat	Size of the vector: 2 × 32, Number of transition tables: 3, Size of each transition table: 32 × 32, Number of generated features: 3072.
Feature selection	INCA	Number of selected feature vectors: 401 (=500 − 100 + 1) Classification accuracy generator: kNN classifier Final feature vector selection method: maximum classification accuracy (greedy algorithm) Length of the final selected feature vector: 174.
Classification	tkNN	Distance metrics: L1 and L2 norms, Weights: Inverse, Equal, and Squared inverse. k values: 1–10, Number of generated parameter-based outcomes: 60, Validation methods: 10-fold CV and LORO CV, Number of generated voted outcomes: 58, Total outcomes: 118, Voting function: iterative majority voting (IMV), Selection of the final feature vector: outcome with maximum classification accuracy (greedy algorithm), Parameters of IMV: Ordering: classification accuracy in descending order, Voting function: mode, Loop range: from 3 to 60, Number of generated outcomes: 58 (=60 − 3 + 1).
XAI	DLob	Number of unique DLob symbols used: 15, Length of the generated DLob string: 446 DLob symbols, Statistical methods: transition table and information entropy.

**Table 3:** The classification results (%) of the introduced ZPat-based XFE model.

Metric	10-Fold CV	LORO CV
Accuracy	99.95	96.12
Sensitivity/recall	99.85	97.11
Specificity	100	95.63
Precision	100	91.61
F1 score	99.93	94.28
Geometric mean	99.93	96.37

**Table 4:** The comparative results.

Research	Method	Type of Data Used	Sample Size	Results (%)
Ko et al. (2022) [52]	EEG Time Series Conversion; Gramian Angular Field (GAF); Recurrence Plot (RP); CNN	EEG (9 channels, N100 stimulus)	81 participants (49 SZH, 32 controls)	GAF: accuracy 93.20, sensitivity 93.90, specificity 92.10 RP: accuracy 90.00, sensitivity 90.9, specificity 88.60
Nsugbe et al. (2022) [53]	EEG; Spectrogram and Scalogram transformations; CNN (SqueezeNet, AlexNet, ResNet18); Handcrafted Features	EEG (60 s, 128 Hz)	20 participants (10 SZH, 10 controls)	Accuracy: 98.30
Benli et al. (2023) [54]	ciSSA (Circulant Singular Spectrum Analysis), SVM, ANN, Ensemble Methods	Resting-state EEG (64 channels, 3 min, 250 Hz)	138 participants (78 first episode psychosis, 60 controls)	Accuracy: 96.23, recall: 96.60, specificity: 95.60, F1 Score: 96.66
Redwan et al. (2024) [55]	ML methods (Random Forest, GPC, SVM, MLP, AdaBoost); PSD analysis	Resting-state EEG signals	72 subjects (44 first episode psychosis patients, 28 controls)	Accuracy: 95.51, recall: 95.30, Specificity: 95.78, F1 Score: 95.26
Ravan et al. (2024) [56]	Resting-state EEG, ReLORETA, Deep Learning Algorithms (3D CNN)	Resting EEG (256 Hz, 10 20 electrode positions)	409 participants (105 MDD, 27 MDD A, 35 MDD P, 71 BD DE, 49 BD ME, 122 SCZ, 239 healthy controls)	Average accuracy: 94.17 (between HC and psychiatric disorders); 93.99 (pairwise classification among disorders).
Our method	ZPat-based XFE Model, INCA Feature Selection, tkNN Classifier, DLob Explainable AI	EEG (32 channel, 15 s segments, 256 Hz)	64 participants (27 psychotic criminals, 37 controls)	(10-fold CV), Accuracy: 99.95 (LORO CV). Accuracy: 96.12

## Data Availability

The authors are committed to making the data available if requested by the journal.
